# Animal Castration

**Published:** 1884-07

**Authors:** 


Animal Castration.—We have seen advance sheets of the opening chapters of a work with the above title by Prof. Liautard, of the American Veterinary College. They are well written, finely illustrated and give promise of a book which will be of the greatest value to Veterinary Surgeons. It is in the hands of W. R. Jenkins the publisher, and will be ready in August. Price $2.00. We will make an extended notice of it in our next number. C.

